# Prevalence and socio-demographic correlates of time spent cooking by adults in the 2005 UK Time Use Survey. Cross-sectional analysis^☆^

**DOI:** 10.1016/j.appet.2015.05.022

**Published:** 2015-09-01

**Authors:** Jean Adams, Martin White

**Affiliations:** Centre for Activity and Diet Research, MRC Epidemiology Unit, School of Clinical Medicine, University of Cambridge, Box 285 Institute of Metabolic Science, Cambridge Biomedical Campus, Cambridge CB2 0QQ, UK

**Keywords:** Cooking, Diet, Nutrition, Time-use, Socioeconomic

## Abstract

This study aimed to document the prevalence and socio-demographic correlates of time spent cooking by adults in the 2005 UK Time-Use Survey. Respondents reported their main activities, in 10 minute slots, throughout one 24 hour period. Activities were coded into 30 pre-defined codes, including ‘cooking, washing up’. Four measures of time spent cooking were calculated: any time spent cooking, 30 continuous minutes spent cooking, total time spent cooking, and longest continuous time spent cooking. Socio-demographic correlates were: age, employment, social class, education, and number of adults and children in the household. Analyses were stratified by gender. Data from 4214 participants were included. 85% of women and 60% of men spent any time cooking; 60% of women and 33% of men spent 30 continuous minutes cooking. Amongst women, older age, not being in employment, lower social class, greater education, and living with other adults or children were positively associated with time cooking. Few differences in time spent cooking were seen in men. Socio-economic differences in time spent cooking may have been overstated as a determinant of socio-economic differences in diet, overweight and obesity. Gender was a stronger determinant of time spent cooking than other socio-demographic variables.

## Highlights

• 85% of women and 60% of men in the UK spent any time cooking over one 24 hour period.
• Median time spent cooking over 24 hours was 50 minutes in women and 10 minutes in men.
• Older, not employed, lower social class, more educated, women spent more time cooking.
• Socio-demographic factors were not associated with time spent cooking in men.

## Introduction

Overweight and obesity are now endemic in many countries (Ng et al., 2014), with socio-economic inequalities disadvantaging the least affluent often seen– particularly in developed countries and women (Friel & Broom, 2007). Unhealthy dietary patterns are part of the complex causal web of overweight and obesity (Butland et al., 2007).

Decreasing home-cooking skills and increasing socio-economic differences in such skills have been proposed as an explanation for increasingly unhealthy diets, rising overweight and obesity, and socio-economic inequalities in these (Lichtenstein & Ludwig, 2010). This is reflected in recent policy focuses on teaching cooking skills. In England, cooking and nutrition were recently re-introduced as mandatory components of the primary school curriculum (Department for Education, 2013); and government-commissioned research is exploring the benefits of cooking skills interventions (Adams, Simpson, Penn, Adamson, & White, 2011; Rees, Hinds, Dickson, O'Mara-Eves, & Thomas, 2012). Poorer cooking skills, less frequent preparation of home-cooked food, and more frequent consumption of pre-prepared foods have been associated with poorer dietary quality and overweight and obesity in observational studies (Hartmann, Dohle, & Siegrist, 2013; Larson, Perry, Story, & Neumark-Sztainer, 2006; Laska, Larson, Neumark-Sztainer, & Story, 2012; McLaughlin, Tarasuk, & Kreiger, 2003; Nelson, Erens, Bates, Church, & Boshier, 2007; van der Horst, Brunner, & Siegrist, 2011; Wolfson & Bleich, 2015). However, there is so far an absence of high quality, definitive, experimental evidence on the impact of cooking skills education on diet or body composition (Rees et al., 2012; Reicks, Trofholz, Stang, & Laska, 2014).

Measuring cooking skills is difficult (Barton, Wrieden, & Anderson, 2011), not least because the concept is complex and there is little agreement about what exactly should be measured (Short, 2003). Possessing cooking skills may also be unrelated to everyday use of such skills. Nevertheless, one UK survey, now almost 20 years old, found little clear evidence of educational differences in self-reported confidence in cooking a range of foods (Caraher, Dixon, Lang, & Carr-Hill, 1999). More recent data from the UK reported that cooking confidence were universally high in low-income households and that over 80% of the low-income population lived in a household where the ‘main food provider’ had well developed cooking skills (Nelson et al., 2007).

One alternative to measuring cooking skills is to measure time spent cooking. Time-use surveys ask respondents to record and account for all of their time (in short episodes) over the course of one or more days. By avoiding focus on any particular activity or behaviour, these surveys may reduce social-desirability bias and provide useful information on a range of activities (Tudor-Locke et al., 2007).

Previous time-use research on time spent cooking has been conducted in the USA (Cawley & Liu, 2012; Kolodinsky & Goldstein, 2011; Mancino & Newman, 2007; Rose, 2007; Smith, Ng, & Popkin, 2013; Zick & Stevens, 2010; Zick, Stevens, & Bryant, 2011), Germany (Moser, 2010) and the UK (Cheng, Olsen, Southerton, & Warde, 2007; Sullivan, 2000). This confirms decreases in time spent cooking since the mid-twentieth century, particularly in women, offset a little by increases in time spent cooking in men. However, women continue to spend substantially more time cooking than men, and more women spend any time cooking than men. Despite noting this, we are aware of only one study in which sex-specific analyses of time spent cooking was conducted (Mancino & Newman, 2007). In the UK, married participants and those with children at home spent more time cooking (Cheng et al., 2007).

There is also conflicting evidence on the socio-economic patterning of time spent cooking. Whilst living in a lower income household is associated with more time spent cooking (Mancino & Newman, 2007; Rose, 2007), female employment is associated with less (Cawley & Liu, 2012; Cheng et al., 2007; Virudachalam, Long, Harhay, Polsky, & Feudtner, 2014).

Time-use and cooking have changed markedly over the twentieth century and are likely to be strongly culturally influenced. It cannot, therefore, be assumed that results from one time or country are generalisable to another. No recent analyses have explored time spent cooking, or its socio-demographic correlates, in the UK. Our aim was to describe the prevalence and socio-demographic correlates of a number of markers of time spent cooking in the UK 2005 Time-Use Survey – the most recent time-use data available from the UK. These, albeit now somewhat historical, data provide the ‘best available’, recent evidence on time spent cooking in the UK.

## Methods

### Data source

The National Statistics Omnibus Survey is a monthly, multi-purpose, cross-sectional survey of UK adults. Data are collected by trained interviewers during home visits. Each month interviewers visit a random probability sample of private addresses selected from the Royal Mail's Postcode Address File of ‘small users’. This is the database of private households in the UK that receive fewer than 50 items of mail per day. It is updated every three months. Sampling is in two stages. Firstly postcode sectors are sampled proportion to their size (that is the total number of addresses contained within each sector); then addresses are selected within sectors at random (Lader, Short, & Gershuny, 2006).

Advance letters are sent to selected addresses explaining the purpose of the survey and stating that an interviewer will visit in the next few weeks. After excluding ‘ineligible’ addresses where there are no current residents, interviewers visit selected households up to eight times at different times of the day and week before coding a household as non-contactable. In contactable households, one resident from all those aged 16 years or over is randomly selected for interview and inclusion in the survey (Lader et al., 2006).

In February, June, September and November 2005, an interviewer administered time-use module was included in the Omnibus Survey. Respondents recalled their main activities in 10-min slots over one 24-h period up to three days prior to the interview – allocated to ensure all days of the week were equally represented (Lader et al., 2006). Together, these four surveys comprise the 2005 UK Time-Use Survey. The original intention of this survey was to answer the question ‘how do we spend our time?’ It is, therefore, multipurpose and data obtained are useful to answer a range of questions. Main activities in each time slot were assigned, at interview, to one of 30 predefined codes, including “cooking, washing up” – referred to throughout as ‘cooking’.

### Variables of interest

#### Time spent cooking

Four measures of time spent cooking were calculated. Total time spent cooking was calculated from the number of 10 minute slots where “cooking, washing up” was reported by respondents as their main activity. This was used to determine whether any cooking was engaged in or not. Longest continuous time spent cooking was calculated from the number of consecutive 10 min slots where this was the main activity. Based on range of recent, popular, recipe books promoting the concept of ‘30 minute meals’ to home, amateur cooks (Lawson, 2013; Oliver, 2010; Pascal, 2012; Slater, 2006), it was assumed that it takes an absolute minimum of 30 minutes to prepare a main meal and longest continuous time spent cooking was dichotomised into spending at least 30 continuous minutes cooking or not.

#### Socio-demographic variables

Socio-demographic variables considered were gender, age (in 10 year age groups), employment status (in paid employment or not), social class, education, and number of adults and children living in the household.

Occupational social class was classified using the National Statistics Socio-economic Classification (NS-SEC) collapsed into three groups (higher and managerial, intermediate, and routine and manual occupations) with those not currently in employment classified according to their last main occupation, or that of the head of household if no last main occupation was available (Rose, Pevalin, & O‘Reilly, 2005). Age at leaving full time education was recorded as younger than 15 years (below school leaving age), between 15 and 18 years (school leaving age) and older than 18 years (post-school leaving age). Number of adults living in the household was dichotomised into one adult, and two or more adults. Number of children living in the household was dichotomised into no children (aged 15 years or younger), and one or more children. These dichotomies allowed the questions of whether living with other adults, or with any children, had any influence on cooking patterns to be explored. For instance, adults may share responsibility for cooking, and the presence of children may be associated with cooking becoming a higher priority.

### Statistical analysis

Complete-case analyses were performed with all analyses restricted to those who provided full data on all variables of interest. As many socio-economic variables are unstable in early adulthood, individuals aged younger than 25 years were excluded from the analysis.

The association between socio-demographic variables and each measure of time spent cooking, after mutual adjustment for all other variables, was explored using separate regression models. Multiple logistic regression was used for the dichotomous outcome variables (any time spent cooking, and at least 30 minutes spent cooking). Multiple linear regression was used for the continuous outcome variables (total time spent cooking, and longest continuous time spent cooking).

As gender acted as an effect modifier of the relationship between measures of time spent cooking and socio-demographic variables in many instances (data not shown), all analyses were conducted separately for men and women. Analyses were performed in Stata v11.

Survey weights are provided with the UK 2005 Time Use Survey data that take account of the unequal probability of individuals within households being selected to take part, as well as adjustments to compensate for differential non-response to the Time Use Survey between different socio-demographic groups, and to ensure that all days of the week were equally represented (Lader et al., 2006). These weights were used in all analyses.

This analysis of anonymised secondary data did not require ethical permission.

## Results

Of 9040 addresses selected for inclusion in the UK Time Use Survey 2005, 717 (8%) were ineligible. Of the remaining 8323 addresses, interviews were achieved with respondents at 5443 addresses (65%). Of these, 4781 (88%) completed full time use diaries. A total of 402 of these individual were aged less than 25 years and so did not meet the inclusion criteria, leaving 4379 (92%) eligible for inclusion in the analyses. Complete data for analyses were available from 4214 participants (96% of those eligible for inclusion in the analyses) – 2292 women and 1922 men.

Tables 1 and 2 summarise time spent cooking overall and by socio-demographic variables, for women and men respectively. In total, 85% of women reported any time cooking and 60% reported at least 30 continuous minutes. Comparable figures for men were 60% and 33%. Women spent a median of 50 minutes cooking and the median longest continuous time was 30 minutes. In men, both figures were 10 minutes.

Tables 3 and 4 show summaries of regression models exploring mutually adjusted socio-demographic correlates of time spent cooking, for women and men respectively. Older women were more likely to report more time cooking according to all four measures. Women in employment spent less time cooking overall, and less continuous time cooking than women who were not employed. Women in the managerial and professional class were less likely to take part in any, and 30 continuous minutes of, cooking than those in the routine and manual class. In contrast, more educated women tended to spend more time cooking. Women living in households with more than one adult, or any children, spent more time cooking according to all measures than those living without other adults or children.

Few consistent socio-demographic differences in time spent cooking were seen in men. Men in employment were less likely to spend any time cooking and their longest continuous time cooking was shorter than men not in employment. Men living in households with more than one adult spent less time cooking according to all measures. In contrast, men living in households with children were more likely to spend 30 continuous minutes cooking than other men.

## Discussion

### Summary of results

This is the most recent study we are aware of exploring prevalence and socio-demographic correlates of time spent cooking in the UK. Despite the data now being ten years old, our results provide the best-available, recent evidence on time spent cooking in the UK. We found clear differences between men and women in prevalence and correlates of time spent cooking. Whilst the great majority of women spent some time cooking, less than two thirds of men did. Overall, almost two thirds of women and one third of men spent at least 30 continuous minutes cooking – our estimate of the absolute minimum time required to prepare a main meal. Employed women and those in more affluent occupational social classes tended to spend less time cooking, whilst women with more education tended to spend more time cooking. Women who lived with other adults and children also tended to spend more time cooking. Few socio-demographic trends in time spent cooking were seen in men.

### Strengths and limitations

The UK 2005 Time-Use Survey is the most recent time-use data available from the UK. However, it is now almost 10 years old. It is not clear how absolute and relative differences in time spent cooking may have changed since 2005. However, there have been some significant changes in food and nutrition practice and culture over the last decade. These include an increasing prevalence of obesity, at the same time as rising consumption of fruit and vegetables (Anonymous, 2012), as well as perceived increases in nutritional awareness and knowledge that are hard to substantiate. Changes have also occurred since 2005 in UK policies and regulations related to food; for example: television marketing of less healthy to children was restricted in 2007 (Adams, Tyrrell, Adamson, & White, 2012). It is difficult to predict how these changes might have impacted on the results reported here. In order to track changes in time-use in general, and time spent on cooking in particular, more regular national time-use surveys would be valuable. Despite these limitations, this is the best available recent evidence on time spent cooking in the UK.

The use of a retrospective, interviewer-administered, pre-coded time use diary may introduce error compared to a prospective, self-completed diary. In 2000–01, a preliminary version of the retrospective, interviewer-administered, pre-coded time use diary used in the current work was piloted at the same time that a prospective, self-completed, free-text time use diary was also in the field. Results were compared to draw conclusions about the validity of the interviewer-administered, pre-coded diary. It was concluded that the interviewer-administered, pre-coded diary allowed a more representative sample of participants to take part, by not excluding those unable or unwilling to engage with a self-completion diary. In particular, retired people and those in poor health were more likely to take part in the pre-coded diary. Overall, estimates of time use between the two different approaches were comparable, but it was recommended that survey weights were applied to the Time-Use Survey (as done here) in order to increase population-representativeness (Lader et al., 2006). Retrospective diaries are also recognised as an appropriate method of time-use data collection by the United Nations (Department of Economic and Social Affairs: Statistics Division, 2005). For these reasons, we believe the time use diary used in the current work was a valid method of assessing time use.

The data used here reflect participants' reports of their ‘primary’ activity in each 10-minute slot throughout the day. Cooking activity lasting less than 10 minutes may not have been captured and our estimates of total time spent cooking are likely to be an underestimate. Individuals have also been observed ‘multi-tasking’ whilst cooking, particularly combining cooking with supervising children (Jabs et al., 2007; Short, 2003). Participants in the UK 2005 Time-Use Survey made their own decisions about what to report as the ‘primary’ activity in these cases and there may have been inconsistency in this, leading to error.

The UK 2005 Time-Use Survey asked participants to allocate time to one of 30 pre-defined activities. The activity used in this work was ‘cooking, washing up’. Whilst we have labelled this ‘cooking’ throughout, it is likely that our calculation of time spent cooking is an over-estimate of time devoted to cooking.

The use of a 30-minute cut-off is arbitrary. Our assumption that a minimum of 30 continuous minutes is required to prepare a main meal is based on recent popular celebrity chef recipe books describing a range of meals that can be prepared by amateur cooks in 30 minutes or less (Lawson, 2013; Oliver, 2010; Pascal, 2012; Slater, 2006). Anecdotal evidence, however, suggests that 30 minutes is optimistic for an amateur cook to prepare many of the recipes included in such books (Hayward, Lusher, Smillie, & Frost, 2010), suggesting that 30 minutes represents an absolute minimum for the time required to prepare a main meal. However, it is also possible to spend less than 30 continuous minutes cooking and still serve a home cooked meal – for example, if meals are prepared in batches in advance (Jabs et al., 2007; Short, 2003). Further work is required to establish population-representative estimates of how long it takes to prepare a range of different meals. The results found in relation to socio-demographic correlates of the other measures of time spent cooking (any time, longest continuous time and total time spent cooking) broadly reflected those found in relation to spending at least 30 continuous minutes cooking. Thus, the use of the 30 minute cut-off does not lead to any results not replicated with other measures.

We used time spent cooking as a proxy for cooking skill. Whilst time cooking may be a more objective measure of whether cooking is engaged in than self-reported skill, time spent cooking may cover a wide range of different activities and result in a range of different outputs – from full preparation of a meal from raw ingredients, to the combination of a range of pre-prepared ingredients requiring minimal additional preparation and heating.

Only 57% of those invited to take part in the UK 2005 Time-Use Survey provided usable diaries. This level of attrition is substantial and could introduce bias. We used study weights, provided with the dataset, to correct for, amongst other things, selective non-response to the survey between socio-demographic groups. By restricting our analyses to adults aged 25 years and older, we avoided any potential mis-classification of socio-economic position in young adults still attending higher education, or still reliant on parental households for food preparation.

### Interpretation of findings

We found that gender is a much stronger determinant of time spent cooking than other socio-demographic variables. The finding that women spend more time cooking than men reflects previous work (Cawley & Liu, 2012; Cheng et al., 2007; Lader et al., 2006; Lake et al., 2006; McLaughlin et al., 2003; Moser, 2010; Virudachalam et al., 2014; Zick et al., 2011). Women living in households with other adults also spent more time cooking than those who did not. The opposite relationship was found in men. This suggests that when men and women co-habit, cooking tends to fall to women. The presence of children in the household also had a strong positive influence on time spent cooking in women, but much less so in men. These findings reinforce that women living in traditional families tend to be responsible for cooking (Lake et al., 2006; Virudachalam et al., 2014). Having children in the household was associated with greater time spent cooking in all adults in UK data from 2000, but not 1975, suggesting that the influence of children on time spent cooking may have developed relatively recently.

Whilst gender differences in time spent cooking may not be a problem *per se*, the fact that even working women spend more time cooking than working men may reflect differential time pressures between men and women. Low income working mothers report wanting to prioritise cooking and feeding their children nutritious meals, but finding it hard to do so given the many other time demands in their lives (Jabs et al., 2007). It is possible that there are ‘ceiling effects’ in time spent cooking and that encouragement to cook more is unlikely to have much impact on women. In contrast, interventions targeted at men may be more effective in increasing time spent on home-cooking (Hunt, Gray et al., 2014). However, this remains a point of uncertainty and the differential effects on individuals, and their households, of delivering cooking skills interventions to men versus women remain unknown. Similarly, given current uncertainty on the effect of cooking skills education on dietary intake and consumption (Rees et al., 2012; Reicks et al., 2014), it cannot be assumed that such interventions will necessarily result in improved health.

The relationship between different measures of SEP and time spent cooking in women reflect previous findings (Cawley & Liu, 2012; Cheng et al., 2007; Moser, 2010). In women, whilst employment and higher occupational social class tended to be associated with less time spent cooking, greater educational attainment tended to be associated with more time spent cooking. Although employment status and occupational social class are often used as measures of affluence, in our models where other markers of SEP were mutually adjusted for, these may more accurately reflect a measure of time available for other tasks, rather than affluence (Cheng et al., 2007). In particular, employed women in this cohort working in more affluent occupations spent more time working than those working in less affluent occupations (data not shown).

Previous authors have suggested that dietary knowledge and interest in cooking may be more important determinants of home cooking than absolute skill (Darmon & Drewnowski, 2008). Greater educational attainment may improve people's ability to gain nutritional knowledge and cooking skills from books and other sources, and increase confidence in one's ability to do so.

Although some authors have reported socio-economic trends in time spent cooking in men as well as women, only Mancino and Newman (2007) have conducted comprehensive stratified analyses (Smith et al., 2013). Like us, they found few strong socio-demographic correlates of time spent cooking in men, but many more in women. This may, in part, reflect the lower absolute prevalence of cooking in men meaning there is less opportunity for variation in men than women.

### Implications of findings for research, policy and practice

Our reliance on time-use data that are more than 10 years old highlights the lack of available data on cooking in the UK. Although both the UK Low Income Diet and Nutrition Survey (Nelson et al., 2007) and the 2008–09 National Diet and Nutrition Survey (Bates, Lennox, & Swan, 2010) collected information on cooking skill and confidence, results from the former suggest that almost all respondents had very high skill (Nelson et al., 2007). Further consideration of what cooking skill is, how to measure it, and studies of population prevalence is required both to identify population groups where further interventions could be targeted, and evaluation of such interventions.

Our findings of strong gender differences in time spent cooking suggest that cooking skills interventions may be most effective if targeted towards men. Recent research promoting dietary knowledge in men has been successful in improving dietary intake and body composition (Hunt, Wyke et al., 2014). However, this remains a point of uncertainty as there is currently an absence of evidence on the effect of cooking skills education on dietary intake and body composition (Rees et al., 2012). But observational evidence does suggest that poorer cooking skills, less frequent preparation of home-cooked food, and more frequent consumption of pre-prepared foods are associated with poorer dietary quality and overweight and obesity (Hartmann et al., 2013; Larson et al., 2006; Laska et al., 2012; McLaughlin et al., 2003; Nelson et al., 2007; van der Horst et al., 2011; Wolfson & Bleich, 2015).

Our finding of inconsistent and weak socio-economic trends in time spent cooking – in women only – suggests that socio-economic differences in home cooking may not be important determinants of socio-economic differences in dietary quality or overweight and obesity. Further research is required to confirm that targeting cooking skills interventions at lower socio-economic groups is an effective way to decrease socio-economic inequalities in diet and body composition.

### Conclusions

In a UK sample, five-sixths of women and almost two-thirds of men spent any time cooking in one 24 hour period. Almost two thirds of women and one third of men spent at least 30 continuous minutes cooking. Gender was a stronger determinant of time spent cooking than other socio-demographic variables. Amongst women, older age, not being in employment, lower social class, greater education, and living in a household with other adults or children were all associated with spending more time cooking. Few differences in time spent cooking were seen in men. Socio-economic differences in time spent cooking may have been overstated as a determinant of socio-economic differences in diet, overweight and obesity.

## Figures and Tables

**Table 1:** Time spent cooking, UK Time-Use Survey 2005; women (n = 2292).

Variable	Level	Sample, %	Time spent cooking or washing up in one 24 hour period			
			Any, % (95% CI)	30 continuous min, % (95% CI)	Total, median (IQR)	Longest continuous, median (IQR)
All women		100	85.0 (83.2 to 86.6)	59.5 (57.3 to 61.8)	50 (20–90)	30 (10–50)
Age group	25–34 years	17.7	81.0 (76.7 to 85.3)	55.4 (49.8 to 61.0)	40 (10–70)	30 (10–40)
	35–44 years	21.8	83.0 (79.2 to 86.9)	60.7 (55.8 to 65.6)	50 (20–90)	30 (10–60)
	45–54 years	19.0	84.5 (80.4 to 88.6)	54.4 (48.6 to 60.0)	40 (10–80)	30 (10–40)
	55–64 years	17.3	89.4 (86.3 to 92.5)	61.0 (55.7 to 66.2)	50 (30–90)	30 (20–50)
	65+ years	24.1	86.8 (83.6 to 90.0)	64.6 (60.4 to 68.8)	60 (30–100)	30 (20–50)
Employment status	Not in employment	45.3	87.2 (85.0 to 89.4)	64.6 (61.6 to 67.7)	60 (30–100)	30 (20–50)
	In employment	54.7	83.1 (80.6 to 85.6)	55.3 (52.0 to 58.6)	40 (10–70)	30 (10–40)
NS-SEC	Routine and manual	41.6	88.1 (85.8 to 90.3)	64.5 (61.1 to 67.8)	60 (30–90)	30 (20–50)
	Intermediate	25.2	85.3 (82.0 to 88.5)	59.6 (55.1 to 64.1)	50 (20–80)	30 (10–40)
	Managerial and prof.	33.2	80.8 (77.5 to 84.2)	53.3 (49.1 to 57.5)	40 (10–80)	30 (10–40)
Age left education	<15 years	11.0	83.7 (78.7 to 88.7)	59.3 (52.9 to 65.7)	50 (20–90)	30 (10–50)
	15–18 years	65.2	86.3 (84.3 to 88.3)	60.1 (57.4 to 62.9)	50 (20–90)	30 (10–50)
	>18 years	23.8	82.0 (78.2 to 85.7)	58.0 (53.1 to 63.0)	40 (20–90)	30 (10–50)
Adults in household	One adult	24.7	82.4 (79.7 to 85.0)	52.4 (49.0 to 55.8)	40 (10–70)	30 (10–30)
	Two or more adults	75.3	85.8 (83.8 to 87.9)	61.9 (59.1 to 64.7)	50 (20–90)	30 (10–50)
Children in household	No children	69.2	83.8 (81.7 to 85.9)	56.7 (53.9 to 59.5)	50 (20–90)	30 (10–40)
	One or more children	30.8	87.5 (84.7 to 90.3)	65.9 (62.0 to 69.9)	50 (30–90)	30 (20–50)

CI: confidence intervals; IQR: inter-quartile range; NS-SEC: National Statistics Socio-economic Classification.

**Table 2:** Time spent cooking, UK Time-Use Survey 2005; men (n = 1922).

Variable	Level	Sample, %	Time spent cooking or washing up in one 24 hour period			
			Any, % (95% CI)	30 continuous min, % (95% CI)	Total, median (IQR)	Longest continuous, median (IQR)
All men		100	60.4 (57.9 to 62.9)	33.0 (30.7 to 35.4)	10 (0–40)	10 (0–30)
Age group	25–34 years	18.6	58.1 (51.6 to 64.6)	28.6 (22.8 to 34.5)	10 (0–30)	10 (0–30)
	35–44 years	22.9	62.8 (57.5 to 68.1)	34.0 (28.9 to 39.2)	20 (0–50)	10 (0–30)
	45–54 years	19.6	54.1 (48.3 to 59.9)	28.0 (22.9 to 33.1)	10 (0–40)	10 (0–30)
	55–64 years	18.1	59.3 (53.7 to 64.9)	32.0 (26.8 to 37.3)	10 (0–40)	10 (0–30)
	65+ years	20.9	66.9 (62.3 to 71.5)	41.3 (36.6 to 46.0)	30 (0–60)	20 (0–30)
Employment status	Not in employment	30.1	66.9 (62.9 to 70.9)	43.2 (39.1 to 47.3)	30 (0–60)	20 (0–30)
	In employment	69.9	57.7 (54.6 to 60.8)	28.6 (25.8 to 31.5)	10 (0–40)	10 (0–30)
NS-SEC	Routine and manual	38.5	58.3 (54.3 to 63.4)	33.9 (20.1 to 37.7)	20 (0–470)	10 (0–30)
	Intermediate	18.5	60.9 (55.2 to 66.6)	33.3 (28.0 to 38.7)	20 (0–50)	10 (0–30)
	Managerial and prof.	43.0	62.1 (58.3 to 65.9)	32.1 (28.5 to 35.7)	10 (0–40)	10 (0–30)
Age left education	<15 years	9.1	65.7 (58.5 to 73.0)	41.6 (34.3 to 49.0)	30 (0–60)	20 (0–30)
	15–18 years	63.9	58.9 (55.8 to 62.0)	21.5 (28.6 to 34.3)	10 (0–40)	10 (0–30)
	>18 years	27.1	62.2 (57.3 to 67.2)	33.7 (28.9 to 38.6)	10 (0–40)	10 (0–30)
Adults in household	One adult	16.1	73.8 (70.0 to 77.7)	42.1 (37.8 to 46.3)	30 (0–60)	20 (0–30)
	Two or more adults	83.9	57.9 (55.0 to 60.7)	31.3 (28.6 to 33.9)	10 (0–40)	10 (0–30)
Children in household	No children	73.3	60.9 (58.0 to 63.8)	32.7 (30.0 to 35.3)	20 (0–40)	10 (0–30)
	One or more children	26.7	59.2 (54.2 to 64.2)	33.9 (29.1 to 38.7)	10 (0–40)	10 (0–30)

CI: confidence intervals; IQR: inter-quartile range; NS-SEC: National Statistics Socio-economic Classification.

**Table 3:** Mutually adjusted logistic and linear regression models of socio-demographic correlates of time spent cooking, UK Time-Use Survey 2005; women (n = 2292).

Variable	Level	Time spent cooking or washing up in one 24 hour period			
		Any, OR (95% CI)	30 continuous min, OR (95% CI)	Total, coefficient (95% CI)	Longest continuous, coefficient (95% CI)
Age group	25–34 years	Reference	Reference	Reference	Reference
	35–44 years	1.00 (0.67 to 1.50)	1.14 (0.83 to 1.56)	6.28 (−0.69 to 13.25)	5.11 (0.62 to 9.60)
	45–54 years	1.62 (1.01 to 2.59)	1.26 (0.88 to 1.80)	10.24 (2.45 to 18.03)	4.75 (−0.10 to 9.60)
	55–64 years	2.74 (1.66 to 4.53)	1.77 (1.20 to 2.62)	20.74 (12.10 to 29.37)	10.56 (4.87 to 16.24)
	65+ years	2.69 (1.49 to 4.87)	2.47 (1.60 to 3.80)	28.12 (17.72 to 38.52)	14.09 (7.10 to 21.08)
Employment status	Not in employment	Reference	Reference	Reference	Reference
	In employment	0.92 (0.66 to 1.29)	0.79 (0.61 to 1.01)	−14.14 (−20.47 to −7.81)	−5.14 (−9.06 to −1.21)
NS-SEC	Routine and manual	Reference	Reference	Reference	Reference
	Intermediate	0.77 (0.54 to 1.08)	0.81 (0.63 to 1.03)	−7.58 (−13.43 to −1.73)	−2.78 (−6.52 to 0.95)
	Managerial and prof.	0.60 (0.43 to 0.84)	0.62 (0.48 to 0.80)	−6.88 (−13.10 to −0.66)	−2.94 (−6.96 to 1.08)
Age left education	<15 years	Reference	Reference	Reference	Reference
	15–18 years	1.71 (1.06 to 2.79)	1.47 (1.03 to 2.09)	8.63 (−0.77 to 18.02)	5.09 (−0.56 to 10.74)
	>18 years	1.67 (0.94 to 2.98)	1.74 (1.13 to 2.67)	9.37 (−2.03 to 20.78)	8.16 (0.52 to 15.79)
Adults in household	One adult	Reference	Reference	Reference	Reference
	Two or more adults	1.39 (1.05 to 1.84)	1.70 (1.40 to 2.07)	17.61 (12.90 to 22.32)	9.42 (6.42 to 12.43)
Children in household	No children	Reference	Reference	Reference	Reference
	One or more children	2.20 (1.50 to 3.22)	2.08 (1.56 to 2.78)	18.55 (12.16 to 24.93)	9.36 (5.13 to 13.59)

OR: odds ratio; CI: confidence intervals; IQR: inter-quartile range; NS-SEC: National Statistics Socio-economic Classification.

**Table 4:** Mutually adjusted logistic and linear regression models of socio-demographic correlates of time spent cooking, UK Time-Use Survey 2005; men (n = 1922).

Variable	Level	Time spent cooking or washing up in one 24 hour period			
		Any, OR (95% CI)	30 continuous min, OR (95% CI)	Total, coefficient (95% CI)	Longest continuous, coefficient (95% CI)
Age group	25–34 years	Reference	Reference	Reference	Reference
	35–44 years	1.19 (0.84 to 1.70)	1.21 (0.83 to 1.75)	4.83 (−1.87 to 11.53)	1.98 (−2.93 to 6.89)
	45–54 years	0.84 (0.59 to 1.21)	0.95 (0.64 to 1.40)	−1.56 (−7.89 to 4.78)	−2.68 (−7.47 to 2.10)
	55–64 years	1.03 (0.71 to 1.52)	1.10 (0.73 to 1.65)	2.25 (−4.89 to 9.39)	−0.29 (−5.44 to 4.87)
	65+ years	1.14 (0.72 to 1.82)	1.12 (0.71 to 1.78)	5.99 (−2.48 to 14.45)	0.53 (−5.29 to 6.36)
Employment status	Not in employment	Reference	Reference	Reference	Reference
	In employment	0.72 (0.52 to 0.99)	0.50 (0.71 to 1.78)	−10.22 (−15.64 to −4.79)	−5.47 (−8.86 to −2.09)
NS-SEC	Routine and manual	Reference	Reference	Reference	Reference
	Intermediate	1.18 (0.88 to 1.59)	1.05 (0.78 to 1.41)	5.64 (−0.41 to 11.69)	2.34 (−1.47 to 6.15)
	Managerial and prof.	1.26 (0.97 to 1.63)	0.98 (0.74 to 1.28)	1.94 (−2.50 to 6.39)	1.04 (−2.07 to 4.15)
Age left education	<15 years	Reference	Reference	Reference	Reference
	15–18 years	0.95 (0.63 to 1.42)	0.93 (0.63 to 1.36)	−2.74 (−10.66 to 5.17)	−1.08 (−6.43 to 4.27)
	>18 years	1.07 (0.66 to 1.73)	1.15 (0.73 to 1.83)	−2.93 (−11.82 to 5.95)	−0.15 (−6.11 to 5.81)
Adults in household	One adult	Reference	Reference	Reference	Reference
	Two or more adults	0.49 (0.38 to 0.63)	0.65 (0.51 to 0.82)	−9.53 (−13.90 to −5.16)	−4.64 (−7.64 to −1.64)
Children in household	No children	Reference	Reference	Reference	Reference
	One or more children	1.14 (0.85 to 1.52)	1.43 (1.05 to 1.94)	3.50 (−1.67 to 8.67)	2.73 (−0.94 to 6.40)

OR: odds ratio; CI: confidence intervals; IQR: inter-quartile range; NS-SEC: National Statistics Socio-economic Classification.
